# The Combination of Adaptive Convolutional Neural Network and Bag of Visual Words in Automatic Diagnosis of Third Molar Complications on Dental X-Ray Images

**DOI:** 10.3390/diagnostics10040209

**Published:** 2020-04-09

**Authors:** Vo Truong Nhu Ngoc, Agwu Chinedu Agwu, Le Hoang Son, Tran Manh Tuan, Cu Nguyen Giap, Mai Thi Giang Thanh, Hoang Bao Duy, Tran Thi Ngan

**Affiliations:** 1 School of Odonto–Stomatology, Hanoi Medical University, Hanoi 010000, Vietnam; nhungoc@hmu.edu.vn (V.T.N.N.); maithigiangthanh@gmail.com (M.T.G.T.); hoangbaoduy@hmu.edu.vn (H.B.D.); 2 Information and Communication Technology Department, University of Science and Technology, Hanoi 010000, Vietnam; agwu.chinedu@gmail.com; 3 VNU Information Technology Institute, Vietnam National University, Hanoi 010000, Vietnam; sonlh@vnu.edu.vn; 4 Faculty of Information Technology, Thuyloi University, 175 Tay Son, Dong Da, Hanoi 010000, Vietnam; tmtuan@tlu.edu.vn; 5 Faculty of Management Information System & E-commerce, Thuongmai University, Hanoi 010000, Vietnam; cunguyengiap@tmu.edu.vn

**Keywords:** dental defect recognition, BoVW, radiology, Adaptive Convolutional Neural Network, dental complications

## Abstract

In dental diagnosis, recognizing tooth complications quickly from radiology (e.g., X-rays) takes highly experienced medical professionals. By using object detection models and algorithms, this work is much easier and needs less experienced medical practitioners to clear their doubts while diagnosing a medical case. In this paper, we propose a dental defect recognition model by the integration of Adaptive Convolution Neural Network and Bag of Visual Word (BoVW). In this model, BoVW is used to save the features extracted from images. After that, a designed Convolutional Neural Network (CNN) model is used to make quality prediction. To evaluate the proposed model, we collected a dataset of radiography images of 447 patients in Hanoi Medical Hospital, Vietnam, with third molar complications. The results of the model suggest accuracy of 84% ± 4%. This accuracy is comparable to that of experienced dentists and radiologists.

## 1. Introduction

Aches from dental defect is one of the worst pains experienced [1] and is difficult to identify or diagnose [2] among people of different age groups. Even though a dentist or radiologist can diagnose dental diseases correctly, there are situations where double confirmations are needed. For instance, in the case where a less experienced dentist or radiologist with a different area of specialty is diagnosing a dental X-ray, a system with the ability to perform object recognition with a high level of accuracy will play an important role in supporting his diagnosis. Another common instance is when the workload of an experienced dentist, who only needs a few seconds to make a diagnosis on one X-ray image, becomes cumbersome when there are a few hundred cases to diagnose, making mistakes inevitable. On the other hand, the automated system can be used for teaching student dentists on performing their housemanship (for educational purposes).

Extensive researches have been done on diagnosing health problems using medical images. For instance, Wu et al. [3] performed diagnosis on periapical lesion using texture analysis and put all texture features into a BoW, while examining all similarities using K-means nearest neighbor classifier. Another example is from Zare et al. [4] who used Scale Invariant Feature Transform (SIFT) [5] and Local Binary Patterns (LBP) [6] as descriptors to build Bag of Visual Word (BoVW), after which images were classified using a Support Vector Machine (SVM). Zare’s method achieved a higher accuracy in putting medical images into various categories. These images in different categories have been made public and used by other researchers. This method is similar to Bouslimi’s method [7]. The major difference is that Bouslimi applied natural language processing for medical images using a dictionary containing while Medical Subject Headings (MeSH) was a controlled dictionary for the purpose of indexing journals, articles, and books in medical and life sciences. It is interesting to note that Zare and Mohammad Reza et al. [8] used a method similar to that of Bouslimi in medical image retrieval, but they suggested using 3 different techniques, which are annotated by binary classification, annotated by probabilistic latent semantic analysis, and annotated by top similar images [7]. All these works are mostly focused in the medical field with radiography images.

There are some other deep learning research works for teeth complication detection. Lee et al. [9] and Li et al. [10] proposed Generating Suspected Tooth-Marked Regions by generating a bounding box for every suspected tooth-marked region. Lee et al. [9] also suggested using Feature Extraction in order to extract feature vectors of the tooth-marked regions (Region on Interests, ROIs) instead of the whole tongue images and finally classification by training a multiple-instance SVM. Lee et al. [11] combined a pre-trained deep Convolutional Neural Network (CNN) architecture and a self-trained network, using periapical radiographic images to determine the optimal CNN algorithm and weights. Pre-trained GoogLeNet Inception v3 CNN network was used for pre-processing the images.

Other studies applied deep learning in medical diagnosis for defective retina (innermost part of the eyes) by Kermany et al. [12] and Rampasek et al. [13] to process medical images and provide an accurate and timely diagnosis of key pathology in each image in return. These images were pre-trained [11,12,13] to reduce the computational time for predicting a result. In cancer detection and diagnosis, Ribli et al. [14] proposed a Computer Aided Design system, which was initially developed to help radiologists analyze screening low-energy X-rays (mammograms). Hu et al. [15] applied a standard R-CNN algorithm to cervical images with long follow-up and rigorously defined pre-cancer endpoints to develop a detection algorithm that can identify cervical pre-cancer. There are quite a few applications of standard CNN to other diseases such as Parkinson disease by Thurston [16], while Rastegari et al. [17] suggested using different machine learning methods including SVM, Random Forest, and Naïve Bayes to apply into different feature sets. In addition to these methods, Similarity Network Analysis (SMA) was performed to validate optimal feature set obtained by using MIGMC technique. To obtain feature standardization, the result of this indicates that standardization could improve all classifiers’ performance.

There are a few notable mentions of BoVW applications outside of the medical community. These applications vary, such as discovering prohibited images by Smith et al. [18]. These items are classified into 6 groups: Gun, Knife, Wrench, Pliers, Scissors, and Hammers. Piñol et al. [19] combined Selective Search with BoVW and CNN for detecting handguns in an airport X-ray image. In summary, there is no established technique for diagnosing dental disease, especially third molar problems. There are research works related to every step of image processing that apply digital image processing methods, traditional classification techniques, and deep learning networks.

In this paper, a dental defect recognition model by the integration of Adaptive Convolution Neural Network and Bag of Visual Word (BoVW) is proposed. The radiologist capturing each image is different, thereby using different radiation dose, angle, focal position, beam filtration, fan angle, etc. This causes important visual features for a clinical diagnosis to be mixed with noisy pixels in the input images. To solve this problem, we apply an image filtering and edge detection technique. First, we use a median filter, and then a bilateral filter. Afterward, we apply the Sobel edge detection technique so that the result image will be more accurate, focusing only on the ROIs rather than irrelevant pixel filled with noises. After noise removal and image smoothing, we set out a ROI.

In this research, we use feature extraction methods namely Scale-Invariant Feature Transform (SIFT) [20] and Speeded Up Robust Features (SURF) [21,22,23] as they give more key-points, which are needed in this case. Since these feature extraction methods do not use the bounding box concept [5,24], we can achieve a bounding box concept by ignoring certain regions of the image using “mask” and specifying X and Y positions on the images. All extracted features are kept in a visual BoVW [23] as unique patterns which can be found in an image. Features are kept in different groups [21,23]. All previous steps are done before feeding the feature into a Neural Network or a SVM for training and afterward prediction.

## 2. Materials and Methods

### 2.1. Materials

The dataset used in this research is originally from the Hanoi Medical University, Vietnam. The dataset included 447 third molar X-ray images, which were classified into three different groups (‘R8_Lower’, ‘R8_Null’, and ‘R8_Upper_Lower’) depending on which part of the dental set was affected by third molars (i.e., by the appearance or how badly tilted they were). All of these images were labeled and sorted by experienced dentists. The dataset was pre-processed by initially converting each image to grayscale, then segmentation, edge detection, and mask ROI before processing by the following four feature extractors: Oriented FAST and Rotated BRIEF (ORB) with BoVW; SURF with BoVW; SIFT with BoVW; and Convolutional Network for Classification and Detection (VGG16). ORB, SURF, and SIFT extracted 2800 features while VGG16 extracted 512 features. We did not train the VGG16 feature extractor because we used a pre-trained model, but in the case of ORB, SURF, and SIFT, we had to train the model using SVM. To train these models, we had to do the following:

1. Key-points and descriptors were extracted by feature extraction technique (i.e., ORB, SIFT, or SURF).

2. All extracted descriptors were clustered into 2800 clusters using K-Means to get visual vocabularies for the BoVW.

3. The visual vocabularies were used to compute the BoVW features from ORB, SIFT, or SURF.

### 2.2. Method

#### 2.2.1. Architecture

**Figure 1:**
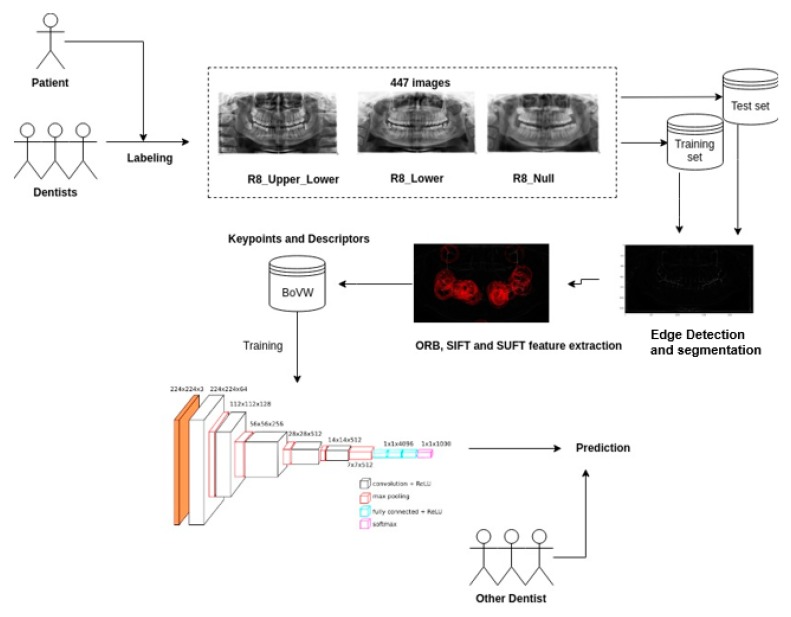
The architecture for third molars’ prediction.

Figure 1 shows the architecture of the procedure in this study. The first step was data gathering step. The dentists provided a properly labeled data set, which consisted of 447 third molar X-ray images taken prior to treatment. The dental experts are from the Hanoi Medical University and are currently working professional dentists.

Next, we applied image filtering and edge detection techniques to smoothen the images and remove unnecessary noise by median filter, and then by a bilateral filter. Afterward, we applied the Sobel edge detection technique. After smoothing the image, we extracted features (ROI)—the third molars—in image pre-processing step. First, ROI, the area affected by third molar complications *t*, was detected by using a mask stating the X and Y location of this defect. Then, we applied SIFT and SURF algorithms to find key points and descriptors of images. All extracted features were saved into a BoVW in order to prepare for training a model. Finally, we fed these BoVW into our designed CNN or an SVM model to make the clinical quality prediction.

**Figure 2:**
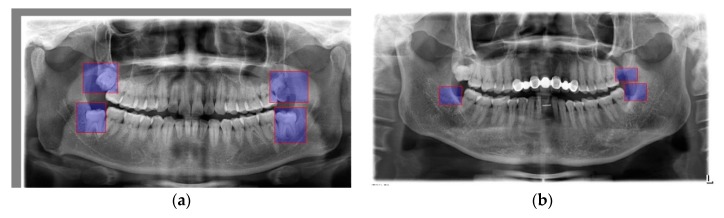
(**a**) Third molar positions; (**b**) treated third molars where blue boxes are the disease parts.

In a typical dental radiography, there are four third molars: two third molars on the upper jaw and two others on the lower jaw. Figure 2a marks out the positions of the third molars with a clear annotation. Figure 2b shows images with missing or treated third molars.

#### 2.2.2. ROI Creation

**Figure 3:**
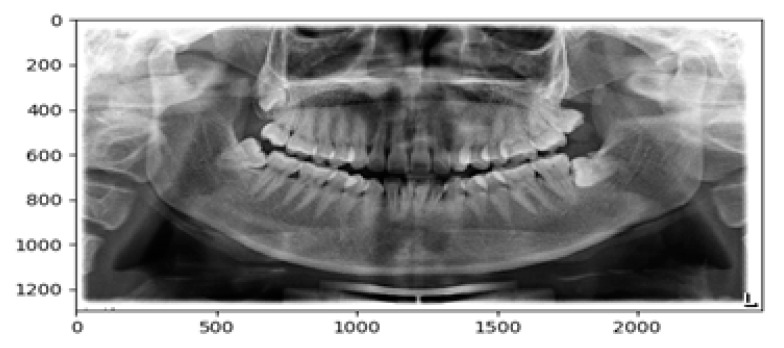
Measuring a dental image where scale bars are measured in pixels.

Since for most human jaw structure and the teeth positions appear at the same area, we can mark out the third molar by specifying their positions using a mask. The mask is not for cropping the image but for specifying which region we would like to examine on the image while ignoring the irrelevant parts. After specifying the mask, we drew the ROI using X and Y coordinates where third molar was usually located. To get this location, we placed the image into a calibrated graph as seen in Figure 3. All third molars must fall within these two regions so that we could specify the coordinates as

{0: rows, 0: cols}

Image {300: 900, 490: 2200}

The change of these figures depends on the image dimension. But for our dataset, these figures are valid.

#### 2.2.3. Image Smoothing

Once we mark out the ROIs, we can proceed to edge detection. The edge detection is required because in the next step we will use a feature detection technique in order to mark key points and because a radiography image displays a lot of unwanted noise that affects the image detection process after that. The edge detection technique’s steps are:

Step 1: Removing noise by blurring most of what might be quantum noise (mottle) with a median filter of kernel size 3.

Step 2: Other noises were removed by bilateral filter with diameter of pixel-9, while Sigma-Color and Sigma-Space were 75 each.

Step 3: Then, we applied Sobel edge detection for both X and Y gradient of the image.

Step 4: Finally, we converted gradients back to uint8, and then blended both gradients into 1.

#### 2.2.4. Feature Extraction

After edge detection, we proceeded to feature extraction. We experimented with multiple feature extraction techniques such as SUFT, SIFT, and ORB [23] because they were faster and accurately detected more features than other feature extraction methods [20]. We got better results with ORB. The feature extraction was applied only to the mask ROI as stated in ROI Creation Section.

#### 2.2.5. Bag of Visual Words

**Figure 4:**
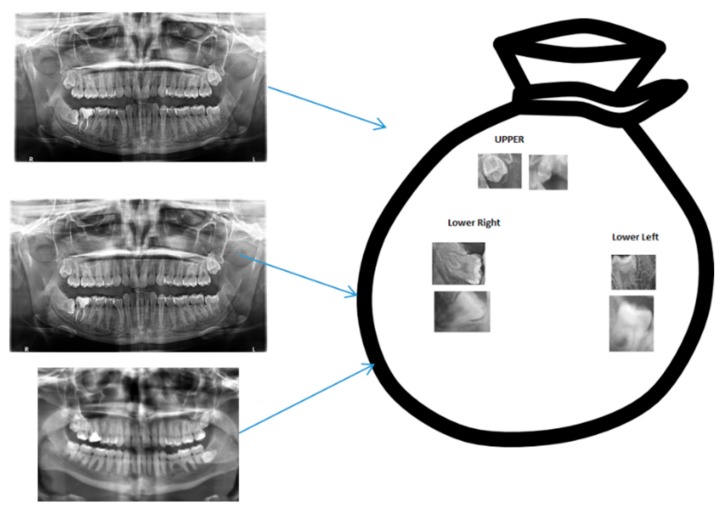
Clustered Key Features into groups where blue arrows imply extraction from the original images to a bag of visual features (words).

A BoVW stores key descriptors frequency of occurrence from the feature extraction phase. A BoVW represents the frequency of descriptors’ occurrence. It does not save the positions of these descriptors but it could save their orientation. In this paper, we created a cluster of these descriptors by K-means clustering algorithm. After clustering, the center of each cluster would be used as the dictionary’s vocabularies. Finally, for each image, we created a histogram from the vocabularies and the frequency of the vocabularies in the image. This histogram was our BoVW. We saved it into a pickle to use in training our SVM/CNN. Figure 4 shows an abstract representation of a BoVW.

#### 2.2.6. Model Training

We train the model using SVM because of its dynamic nature. SVM has some advantages for doing so, for example, the ability against over-fitting [25]. The model is able to handle any number of input space as well. To avoid these high dimensional input spaces, we assume that most of the features are irrelevant. Feature extraction/selection tries to point out these unnecessary features. Any classifier using the worst features will have much better performance than a classifier using random features.

#### 2.2.7. Convolutional Neural Network

**Figure 5:**
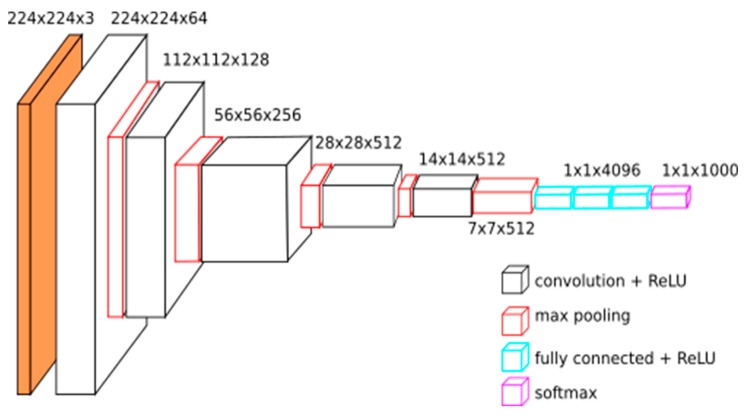
The structure of a Convolutional Neural Network (CNN) [26].

Convolutional neural network (CNN) used in this research contains 16 hidden convolutional layers, a max pooling layer, and fully-connected layers. Figure 5 shows a clear description of a CNN. In this CNN, both convolution and fully-connected layers apply activation function called Rectified Linear Unit (ReLu) while the output layer applies SoftMax activation function to estimate the probability of the various classes. These experiments use TensorFlow-backed Keras as the framework for deep learning and scikit-learn for classification and evaluation. This helps us reduce the amount of boiler plate codes written. This also helps us achieve a faster research work as well.

## 3. Experiments

The experimental objective is to compare the performance of different feature extraction methods (ORB, SIFT, SURF, and VGG16) with multiple classifiers namely:

- Logistic Regression
- Support Vector Machine (RBF Kernel)
- ANN/Multi-Layer Perceptron (1 hidden ReLU layer and 100 nodes)
- Decision Tree
- Gradient Boosting
- Random Forest

**Figure 6:**
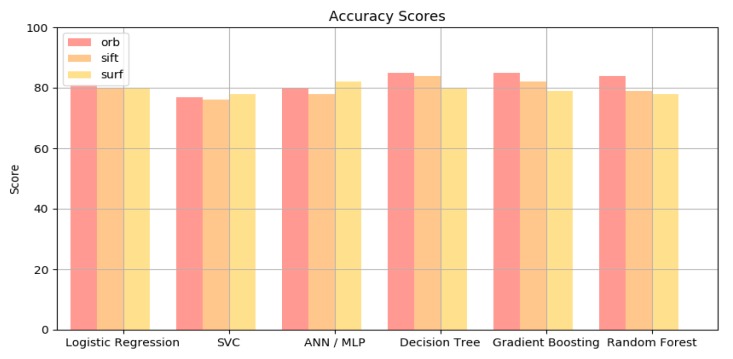
The comparison of accuracy among related methods.

Table 1 shows the results from experiments which were done with 10-fold cross validation method. The number of folds indicates how the dataset is divided. In this case, the dataset is separated into 10 equivalent parts, where one part is chosen to validate against the remaining. This improves the accuracy of the model as a whole. In general, the VGG16 feature extractor performs just as good as ORB, SIFT, and SURF feature extractors because it has just fewer features (512 vs. 2800 features). Since VGG16 does not need training, it is more efficient than ORB, SIF, or SURF BoVW. Furthermore, neural network model such as VGG16 can run parallel in GPU, which gives it an efficiency edge over BoVW extractor. The results in this table show that Decision Tree, ANN/MLP, and Gradient Boosting generally get better results in accuracy comparing to the other classifiers. Note that ORB extractors are also much better than all other extractors. This is due to the fact that ORB has the fastest key points matching and best performance among others such as SIFT and SURF [27]. On the average, accuracy of the proposed model is slightly higher than that of VGG16 as shown by a chart in Figure 6. SVM model completes training in a shorter time which is good for low performance computers, but the prediction usually takes longer than the VGG16 (30 s vs. 5 s, respectively). In real life applications, the time difference may not be so daunting.

## 4. Conclusions

In this paper, we proposed an Adaptive Convolution Neural Network and BoVW for dental defect recognition. In BoVW, we represent an image as a set of features, which consists of key-points and descriptors. Taking a look in the application feature extractions algorithm and BoVW in the medical as well as other branches of science, the possibilities are enormous. We can see that with this model, the prediction can be done quickly and correctly. In the experiments, we collect a dataset of radiography images from 447 patients in Hanoi Medical Hospital, Vietnam, with third molar complications. The results of the proposed model suggest an accuracy of 84% ± 4%. This accuracy is comparable to that of the experienced dentists and radiologists. Miss-classification is caused by the fact that dental images are very closely related in features. This requires some specific methods to overcome this challenge. The upside to this is that image classification with ORB, SIFT, and SURF were good. These descriptors are strong tools when they are applied into dental field, in particular, or medical field, in general. In the combination with SVM classifier, it builds an efficient and dependable system for classification and prediction.

Modified Hopfield neural network [26] or advanced fuzzy decision making [28] would be the solution to our problem, which warrants further research. Moreover, similar to the research in [29,30], the proposed algorithms need to be applied to different datasets. In future works, we will make the implementations on a wider range of different, complex dental image datasets to verify the suitability of this algorithm. Other integrations between deep learning and fuzzy computing in our previous studies [28,31,32] would be vital for favorable outcomes in upcoming research.

## Figures and Tables

**Table 1:** The experimental results by applying different classifiers (notation - stands for “undefined”).

Classifiers	ORB	SURF	SIFT	VGG16
Logistic Regression	81%	80%	80%	-
SVC	77%	76%	78%	-
ANN / MLP	83%	78%	82%	-
Decision Tree	85%	84%	80%	-
Gradient Boosting	85%	82%	79%	-
Random Forest	84%	79%	78%	-
CNN	-	-	-	84%
